# Base Flip in DNA Studied by Molecular Dynamics Simulations of Differently-Oxidized Forms of Methyl-Cytosine

**DOI:** 10.3390/ijms150711799

**Published:** 2014-07-03

**Authors:** Mahdi Bagherpoor Helabad, Natalia Kanaan, Petra Imhof

**Affiliations:** Institute of Theoretical Physics, Free University Berlin, Arnimallee 14, 14195 Berlin, Germany; E-Mails: mbagerpoor@zedat.fu-berlin.de (M.B.H.); kanaan@zedat.fu-berlin.de (N.K.)

**Keywords:** DNA damage, base flip, molecular dynamics simulations, DNA recognition

## Abstract

Distortions in the DNA sequence, such as damage or mispairs, are specifically recognized and processed by DNA repair enzymes. Many repair proteins and, in particular, glycosylases flip the target base out of the DNA helix into the enzyme’s active site. Our molecular dynamics simulations of DNA with intact and damaged (oxidized) methyl-cytosine show that the probability of being flipped is similar for damaged and intact methyl-cytosine. However, the accessibility of the different 5-methyl groups allows direct discrimination of the oxidized forms. Hydrogen-bonded patterns that vary between methyl-cytosine forms carrying a carbonyl oxygen atom are likely to be detected by the repair enzymes and may thus help target site recognition.

## 1. Introduction

The genomic integrity of the cell is constantly threatened by DNA damage, nucleotide changes, deletions or recombinations, or epigenetic modifications, leading to mutations. A complex machinery of interacting DNA processing repair enzymes protects the cell from these distortions. Typical targets of such repair enzymes are abasic sites, damaged or alkylated nucleotides or non-native bases, such as uracil. Glycosylase enzymes recognize mismatches and damage and specifically remove the wrong base. The resulting abasic site is then processed further by other DNA repair enzymes. Preferred sites are CpG sites [1], which are the target for human methyltransferase.

From the many structures of glycosylases complexed to damaged DNA [2,3], it is known that damaged, mispaired or wrong bases are flipped out of the helical DNA duplex into the enzyme’s active site. In the debate on how glycosylase enzymes recognize a damaged or mispaired base, two mechanisms are discussed. One is a passive mechanism in which the enzyme detects extra-helically-exposed, already, at least partially, flipped-out bases. This mechanism implies that base pair opening up to several degrees of flipping is more likely for damaged/mispaired bases than for intact canonical ones. The alternative mechanism involves flipping of the base, while the enzyme travels along the DNA, relying on the enzyme specifically enhancing the flip-out of its target base [4,5].

In addition to the deamination products of 5-methyl-cytosine (mCyt), thymine DNA glycosylase (TDG) has been reported to be involved in active DNA demethylation through the removal of the oxidized derivatives of 5-methyl-cytosine: 5-formyl-cytosine (5fC) and 5-carboxyl-cytosine (5caC) will be recognized and expelled by TDG, following the base excision repair pathway [6,7,8], whereas 5-hydroxymethyl-cytosine (5hmC) and 5-methyl-cytosine (5mC) are not processed by the glycosylase enzyme. Human TDG has been crystallized in complex with DNA containing various mismatches, including G:5caC and G:5hmU (5-hydroxymethyl-uracil). 5caC is also flipped out of the double-stranded DNA into the enzyme’s active site, but exhibits a conformation different from those reported for TDG complexed to substrate analogues [8,9]. The post-reactive complex of TDG with caC did not show any interactions of protein side-chains with the DNA major groove where the methyl groups are located, but, instead, interactions with the phosphate group of the flipped nucleotide [8]. This finding led the authors to suggest that the discrimination between different 5-methyl groups is achieved or at least facilitated by other means of recognition. It has been speculated that, e.g., G:5fC and G:5caC form mismatch-like wobble hydrogen bonding pattern via an amino-imino protonation equilibrium [10,11] that is shifted towards the imino site for formyl and carboxyl cytosine, stabilized by the possibility to form an intra-molecular hydrogen bond to the carbonyl oxygen atom. NMR studies of 5-formyl-2’-deoxycytidine in DMSO and calculations of isolated 5fC and 5caC bases in implicit water, however, suggest that the amino form is energetically more stable and, thus, predominant [12].

Glycosylase enzymes are discussed as following a multi-step interrogation pathway to discriminate their target base from non-cognate ones. Partial distortion of the DNA helix, intra-helical interrogation to detect a lesion and base flipping in varying degrees are thought to ensure that only the substrate-base is processed. Biochemical DNA binding data show binding to C, 5mC and 5hmC to be significantly weaker than binding of substrate bases. This has been interpreted as a discrimination step before base flip and reactive complex formation [7,13]. However, among the possible base substrates analyzed, only thymine and uracil variants would have a non-Watson–Crick hydrogen bonding pattern that is likely to stabilize distorted, wobble pairs or even partially-flipped conformations [14] that are easy to recognize by the repair enzyme.

Molecular simulations have proven to be a powerful tool for obtaining information on the structure and dynamics at the atomic level and have been used successfully to analyze interactions between proteins and DNA.

There is a vast literature on molecular dynamics studies of DNA analyzing the structural and dynamical differences of various damages, lesion or mispairs [15,16,17,18,19,20]. Simulations of base flip have been successfully conducted on free DNA [21,22,23,24,25,26] and in complex with different DNA repair enzymes [26,27,28,29,30,31,32], applying various flavors of enhanced molecular dynamics. The recognition and base flip of cytosine has been studied in a series of molecular dynamics studies [26,30,31]. Huang *et al*. investigated the spontaneous base flip in free DNA in aqueous solution, in a binary complex with *Hha*I methyltransferase, and in a ternary complex containing protein, DNA and the cofactor, *S*-adenosylhomocysteine (SAH). They observed the free energy barrier for the cytosine base flip in uncomplexed DNA to be independent of the flanking sequence. In contrast, when complexed to the methyltransferase and cofactor, the barriers for base flipping have been found to be significantly higher in non-cognate sequences than in cognate DNA.

We have previously shown that DNA containing a single T:G mispair exhibits local dynamics significantly different from DNA without such a mispair. The T:G wobble pair shows a distorted conformation compared to T:A or C:G pairs. Our free energy calculations show that thymine is much more probable to be flipped than cytosine in a C:G pair or thymine in a T:A pair, a fact that can be exploited by the repair enzymes. Moreover, a partially open state of the T:G mispair, which we observe to be transiently occupied also in the unbiased simulations, is supposedly easy to be recognized by the searching repair enzyme [14]. These results suggest that DNA repair enzymes, such as glycosylases, can first recognize local distortions in the base steps and base-pair geometries, which deviate from normal B-form DNA. Those distorted sites are then likely to be further examined by the repair enzyme, including the attempt to flip out the putative mispaired or damaged base into the active site of the repair enzyme.

In the present paper, we analyze the dynamics of DNA containing oxidized and intact cytosine, so as to reveal which structural and dynamical differences might facilitate the discrimination of the target bases for removal (5fC and 5caC) over the very similar C, 5mC and 5hmC bases.

## 2. Results

### 2.1. DNA Conformation

**Figure 1:**
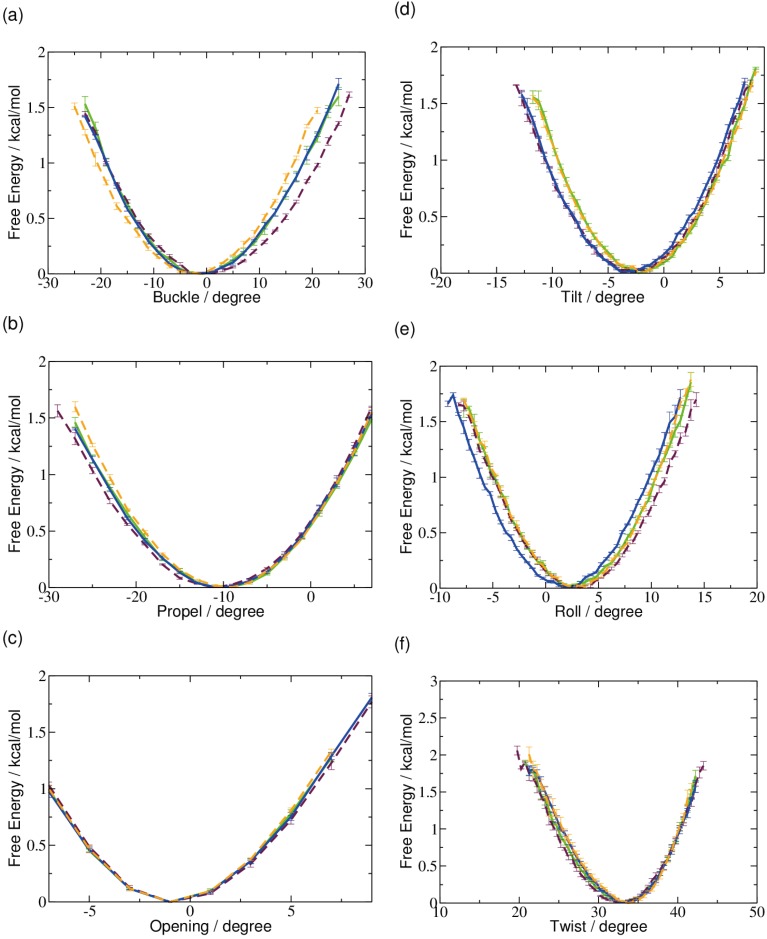
Free energy profiles of local helical rotational parameters of 5-methyl-cytosine (green), 5-hydroxymethyl-cytosine (maroon), 5-formyl-cytosine (blue) and 5-carboxyl­cytosine (orange), respectively: (**a**) buckle; (**b**) propeller twist; (**c**) opening; (**d**) tilt; (**e**) roll; and (**f**) twist.

**Figure 2:**
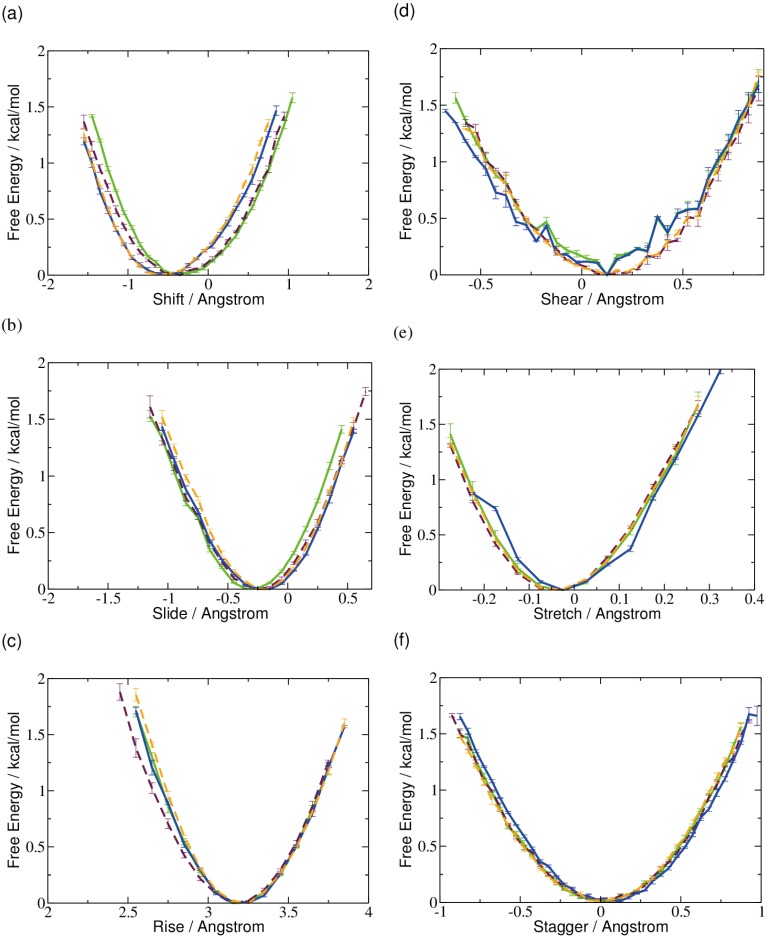
Free energy profiles of local helical translational parameters of 5-methyl-cytosine (green), 5-hydroxymethyl-cytosine (maroon), 5-formyl-cytosine (blue), and 5-carboxyl­cytosine (orange), respectively: (**a**) shift; (**b**) slide; (**c**) rise; (**d**) shear; (**e**) stretch; and (**f**) stagger.

We have examined the conformation of the DNA double helix carrying the different forms of oxidized and intact methyl cytosine, analyzing the local conformation at the central G:Cox pair. Figure 1 and Figure 2 show the free energy profiles of the intra-and inter-base pair parameters involving the methyl cytosine. The free energy minima for all rotational parameters are the same. The only difference between the models is the slightly larger range of tilt and roll angle explored by formyl-cytosine and a similarly slightly larger range of buckle angle sampled by 5-hydroxymethyl-cytosine.

Furthermore, for the translational base-pair and base-step parameters, only marginal differences can be observed between the different forms of methyl cytosine. The free energy profile for the shear parameter is less smooth in the case of native and 5-hydroxymethyl-cytosine compared to the higher oxidized forms. However, both the free energy minimum and range of the shear translation are similar for all types.

### 2.2. Base Flip

**Figure 3:**
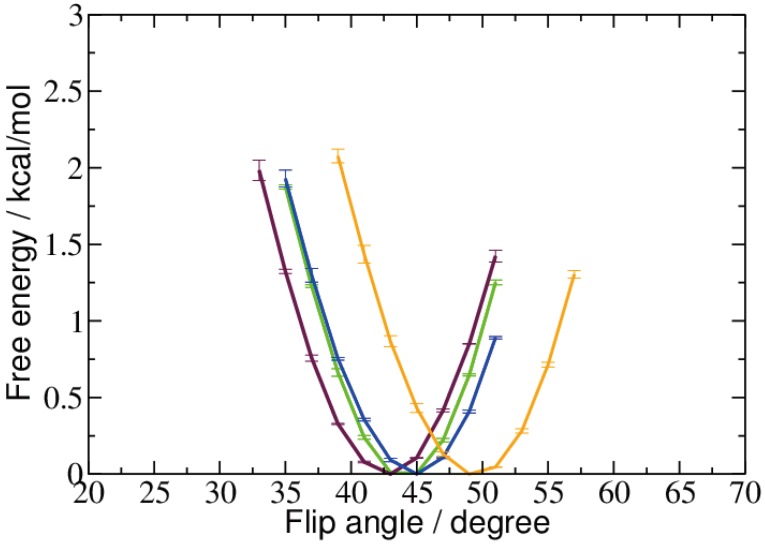
Free energy profiles of the pseudo-dihedral flip angle evaluated from the unbiased MD simulations of 5-methyl-cytosine (green), 5-hydroxymethyl-cytosine (maroon), 5-formyl-cytosine (blue) and 5-carboxyl-cytosine (orange), respectively.

Figure 3 shows the free energy profile of the flip angle around its equilibrium values, computed from the unbiased simulations. Except for the 5-carboxyl-cytosine (minimum at ˜48°), all models exhibit a similar free energy minimum of the flip angle, between ˜42° and ˜46°.

**Figure 4:**
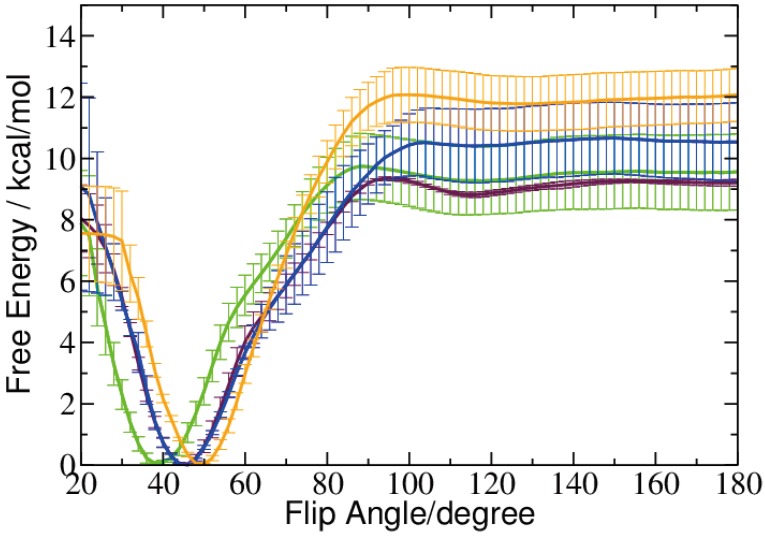
Free energy profile of the base flip for 5-methyl-cytosine (green), 5-hydroxymethyl-cytosine (maroon), 5-formyl-cytosine (blue) and 5-carboxyl-cytosine (orange). The pseudo dihedral coordinate is illustrated in *Section 4.2.2.*

The free energy computed for the rotation (flip) of a single base out of the DNA double helix is plotted in Figure 4. A complete rotation of the base, including the passage of the minor groove, requires very high forces and leads to a deformation of the DNA. We therefore restricted the flip to a rotation through the major groove.

The positions of the free energy minima computed from the biased simulations at ˜48° are virtually the same for 5fC and 5caC. The less oxidized 5-hydroxymethyl-cytosine exhibits a free energy minimum of the flip angle at a slightly smaller value of ˜45°, and the unoxidized methyl-cytosine shows a most probable flip angle at ˜38° (see Figure 3 and Figure 4). This is a shift towards a smaller flip angle by ˜5° compared to the unbiased simulation. For 5hmC, we also observed a small difference in the biased compared to the unbiased simulation, however, this time, towards a larger flip angle.

### 2.3. Hydrogen Bonds and Solvent Accessibility

Table 1 lists the occupancies of the hydrogen bonds in the base pair between the different forms of methyl-cytosine and guanine, as well as hydrogen bonds between the methyl-cytosine base and bulk water. The inter-base pair hydrogen bonds are of similar strength in all models investigated here with a somewhat larger standard deviation in the case of 5-hydroxymethyl-cytosine. Furthermore, the hydrogen-bond interaction between the O2 oxygen atom and bulk water is comparable in all four models. A significant difference, however, can be observed for the hydrogen bonds formed between the N4 nitrogen atom and bulk water: the higher oxidized forms, 5-formyl-and 5-carboxyl-cytosine, show only little occupancy for that hydrogen bond. This can be attributed to the fact that the N4 amino group forms an intra-molecular hydrogen bond with the formyl or carboxyl oxygen atom, respectively. Although an intra-molecular hydrogen bond can, in principle, also be formed between N4 and the hydroxyl oxygen atom, this is not the case, and hence, the interaction between N4 and bulk water appears not to be influenced by the extra hydroxyl group.

**Table 1:** Occupancies of hydrogen bonds between DNA base pairs computed from the sim-ulation of 5-methyl-cytosine (5mC), 5-hydroxymethyl-cytosine (5hmC), 5-formyl-cytosine (5fC) and 5-carboxyl-cytosine (5caC).

Donor	Acceptor	Occupancy/%			
		5mC	5hmC	5fC	5caC
GUA7-N2	CYT7-O2	93.0 ± 1.1	92.2 ± 5.6	94.9 ± 1.2	95.8 ± 0.4
CYT7-N4	GUA7-O6	86.9 ± 0.7	88.8 ± 6.6	87.2 ± 0.4	88.1 ± 0.5
GUA7-N1	CYT7-N3	96.9 ± 0.9	94.7 ± 1.2	95.4 ± 1.0	95.8 ± 0.2
Water	CYT7-O2	73.2 ± 2.8	70.7 ± 0.2	70.7 ± 0.2	73.4 ± 0.5
CYT7-N4	Water	30.2 ± 1.6	32.2 ± 1.1	6.9 ± 0.1	9.4 ± 0.4
OX1	Water		9.8 ± 0.2		
Water	OX1		51.4 ± 0.7	66.1 ± 3.3	95.8 ± 3.3
Water	OX2				88.0 ± 3.2

The hydrogen-bonds between the oxygen atom and bulk water in the two forms carrying one oxygen atom (5hmC and 5fC) are comparably probable, whereas 5-carboxyl-cytosine not only forms hydrogen bonds to bulk water with both oxygen atoms, but also these hydrogen bonds are significantly stronger than those of the other oxidized methyl-cytosine forms.

**Figure 5:**
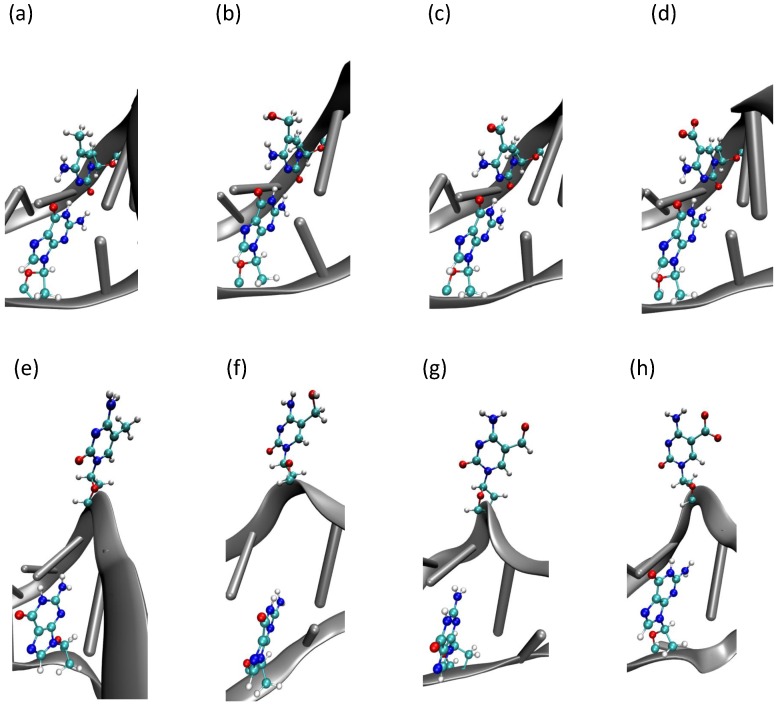
Snapshots of the DNA base flip simulation taken at about the free energy minimum of (**a**) 5-methyl-cytosine; (**b**) 5-hydroxymethyl-cytosine; (**c**) 5-formyl-cytosine; and (**d**) 5-carboxyl-cytosine; and snapshots of the DNA base flip simulation taken at about the free energy maximum of (**e**) 5-methyl-cytosine; (**f**) 5-hydroxymethyl-cytosine; (**g**) 5-formyl-cytosine; and (**h**) 5-carboxyl-cytosine. Colours refer to different atom types: red: oxygen; cyan: carbon; blue: nitrogen; white: hydrogen.

We have furthermore computed the solvent accessible surface area and the radial distribution function of bulk water around the methyl-cytosine bases and the (oxidized) methyl groups. Whereas the solvent accessibility of the entire base is almost the same for all forms of methyl-cytosine investigated, there is a small effect of the methyl group itself. With increasing oxidation of the methyl group, and, hence, also increasing size, the solvent accessible surface area also increases (Table 2). Only in the case of carboxyl-cytosine, also the base exhibits a somewhat larger solvent accessible surface area than the less oxidized forms, which is perfectly explained by the carboxyl group extending to the solvent (Figure 5).

**Table 2:** Solvent accessible surface area (Sasa) of the base and the oxidized methyl group in 5-methyl-cytosine (5mC), 5-hydroxymethyl-cytosine (5hmC), 5-formyl-cytosine (5fC) and 5-carboxyl-cytosine (5caC).

	Sasa/nm^2^			
Group	5mC	5hmC	5fC	5caC
Base	0.691 ± 0.002	0.696 ± 0.003	0.667 ± 0.001	0.709 ± 0.006
Methyl group	0.393 ± 0.002	0.485 ± 0.001	0.471 ± 0.002	0.529 ± 0.004

**Figure 6:**
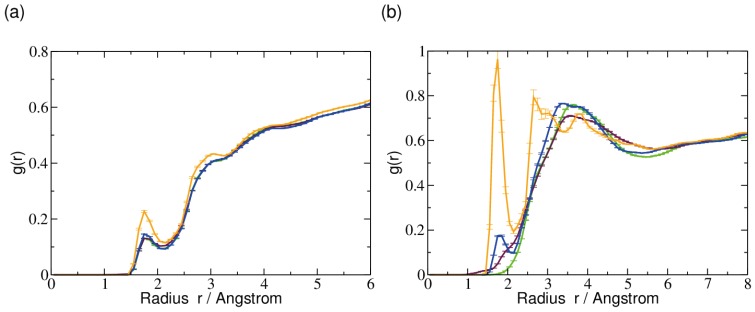
Radial distribution functions *g*(r), with r = Radius, of water surrounding (**a**) the base and (**b**) the (oxidized) methyl groups in the central 5-methyl-cytosine (green), 5-hydroxymethyl-cytosine (maroon), 5-formyl-cytosine (blue) and 5-carboxyl-cytosine (orange).

The water accessibility computed for the differently oxidized methyl cytosine bases as quantified by the radial distribution of water molecules surrounding the base (Figure 6) is again very similar for all forms of methyl cytosine, and again, the carboxylated form is the exception, which shows a higher probability for water molecules to be in the first solvation shell, *i.e.*, at a distance of ˜1.8 Å. At the larger distances, the probability of finding a water molecule agrees in all cases studied, showing that the second, third or higher solvation shells are not affected by the differences in the oxidation level. When analyzing the distribution of water molecules around the methyl groups only, the difference between the carboxylated form and the other ones becomes even more pronounced. Here, a rather large peak can be observed at a distance of ˜1.8 Å from the methyl group, indicating a well-ordered first solvation shell. The significantly larger height of that peak can be attributed to two oxygen atoms as opposed to only one in the other forms that are likely to form hydrogen bonds to bulk water molecules (*cf.*, also, Table 1). Furthermore, 5-formyl-cytosine also shows a peak at that distance, albeit smaller than that of 5-carboxyl-cytosine. 5-hydroxymethyl-cytosine shows a somewhat lower probability for water molecules at that distance from the methyl group and appears to lack any structural order that can be interpreted as a first solvation shell. For 5-methyl-cytosine only at the distance of a second or even third solvation shell, the water distribution becomes significant. This is at a distance at which all three uncharged models are rather similar.

## 3. Discussion

Our molecular dynamics simulations indicate that the conformations and flexibility of DNA carrying oxidized forms of 5-methyl-cytosine (5-hydroxymethyl, 5-formyl and 5-carboxyl) are essentially the same as those of DNA with native 5-methyl-cytosine. All base-pair and base-step parameters investigated differ only marginally for the four methyl-cytosine models.

The pseudo-dihedral angle defining the coordinate for the base flip suggests that all four methyl-cytosine forms are rather unlikely to flip-out spontaneously. The free energy barrier computed for the flip out of the DNA through the major groove is between 9 and 12 kcal/mol, comparable to the free energy barrier calculated for unmethylated cytosine [14,21,30] and significantly higher than the free energy barrier for the base flip of a mispaired thymine, both computed in a previous study [14].

**Figure 7:**
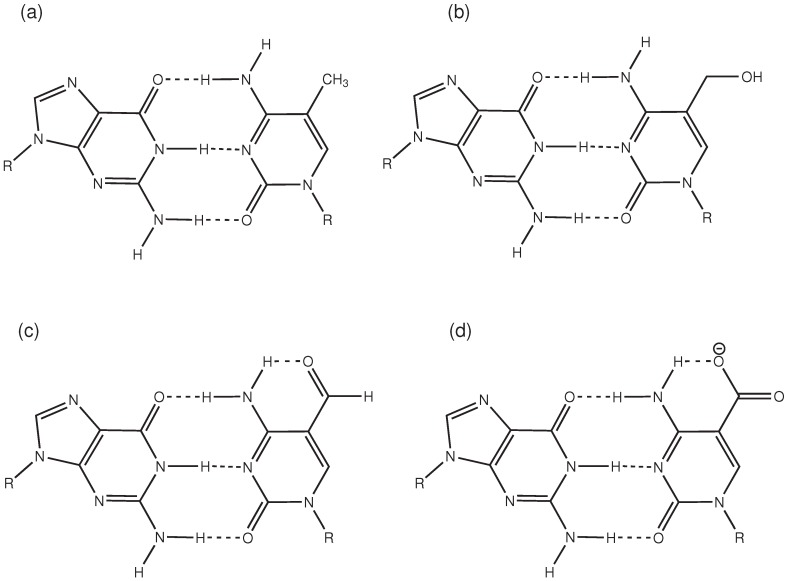
Schematic drawing of (**a**) 5-methyl-cytosine; (**b**) 5-hydroxymethyl-cytosine; (**c**) 5-formyl-cytosine; and (**d**) 5-carboxyl-cytosine.

Our data suggest that the intrinsic probability of the target base for wobble conformations and displacement towards base-pair opening or flip that has been found for thymine is not present in the methyl-cytosine forms investigated here and, therefore, cannot be exploited by the enzyme. However, we cannot rule out the idea that the different equilibria between the amino-imino tautomeric variants (see, e.g., [8], Figure 7) of the oxidized methyl cytosines do play a role in target site recognition [8]. Whereas the NMR studies and calculations of individual methyl cytosines in [12] very convincingly conclude that the amino form is prevailing, the formation of imino-tautomers can still be different in solvated DNA and, therefore, also be more likely (or less unlikely) for formyl and carboxyl-cytosine than for native and 5-hydroxymethyl-cytosine. Following that assumption, formyl and carboxyl-cytosine are also more probable to form “wobble pairs” with guanine that are similar to mismatches in their displaced conformations, as well as in a reduced energy requirement for base flip compared to intact (amino) Watson–Crick base pairs. Simulations of imino forms and calculations of amino-imino equilibria in solvated DNA are subject of an ongoing study.

Another possibility to recognize oxidized methyl-cytosine in their amino forms is by direct contacts between protein and base. In contrast to the rather indirect recognition of distorted wobble pairs whose specific hydrogen bonds are buried in the DNA helix to a large extent, even for partially-flipped conformations, the (oxidized) methyl group is comparably easy to be accessed and probed by protein residues. Our simulation data show that the oxidized forms that are processed by the glycosylase enzyme, formyl and carboxyl, indeed show more hydrogen-bond interactions with bulk water, a higher probability of forming a (structured) first solvation shell and also have a larger solvent accessible surface area. Hence, it is conceivable that the protein can directly access the oxidized site and form specific contacts that might destabilize the Watson–Crick state. That way, discrimination would be achieved by energetically favoring the flip of the target bases only. For the cytosine-specific methyltransferase, *M.Hha*I, such an “energetic recognition mechanism” has been reported in which the enzyme’s specificity depends on the ability to exclusively facilitate flipping of the target base: a lowering of the free energy barrier for base flip is only observed upon the formation of specific protein–DNA interactions, such as hydrogen bonds to the target cytosine [30,31].

An additional discrimination possibility is via the very low probability of the amino group forming hydrogen bonds to bulk water in the 5-formyl and 5-carboxyl forms, as opposed to 5-methyl and 5-hydroxymethyl cytosine. Similarly, hydrogen bonds that can (or cannot) be formed with water can be envisaged to be formed (or not) between the enzyme and the base. As a result, the protein would no longer interrogate a site that allows hydrogen bonds to be formed with its amino group N4 atom.

This amino group is not present in thymine, and hence, contacts as formed between protein and the amino group are lacking here, too. This suggests that direct readout via hydrogen bonds that can be formed in the non-cognate bases as opposed to all bases known to be processed by the repair enzymes is an essential element of target base recognition.

## 4. Experimental Section

### 4.1. Model Setup

DNA oligonucleotides of a 13 base pairs length with a central G:C pair were prepared using web3DNA [33]. Then, four models were constructed in which the central cytosine base has been changed to 5-methyl-cytosine (5mC), 5-hydroxymethyl-cytosine (5hmC), 5-formyl-cytosine (5fC) and 5-carboxyl-cytosine(5caC), respectively (Figure 7).

The systems were solvated with explicit water, using the TIP3Pmodel [34], extending to at least 10 Å beyond the DNA in each direction in a tetragonal box for the unbiased simulations of size (*x* = 90 Å, *y* = *z* = 60 Å) and in a larger cubic box (*x* = *y* = *z* = 90 Å) to allow for flipping of the central base. Twenty four Na^+ ^counter-ions were added to neutralize the system and an excess of Na^+ ^and Cl^−^ ions to obtain a physiological concentration of 150 mM NaCl. The addition of the ions was carried out by the random substitution of water oxygen atoms.

Simulations were performed using periodic boundary conditions, and the long-range electrostatic interactions were treated using the particle mesh Ewald method [35] on a 96 × 60 × 60 charge grid for the unbiased and on a 96 × 96 × 96 charge grid for the biased simulations, respectively. A non-bonded cut-off of 12 Å was applied. The short range electrostatics and van der Waals interactions were truncated at12 Å. A using a switch function starting at 10 Å.

The solvated structures were minimized using 5000 steps of steepest descent, followed by minimization with the conjugate gradient algorithm, with solute atoms harmonically constrained until an energy gradient of 0.01 kcal/(mol·Å) was reached. The system was then gradually heated for 30 ps to 300 K with 1 K temperature steps with harmonic restraints on the solute atoms.

The systems were equilibrated in three different stages with the numbers of particles, pressure (1 bar) and temperature kept constant (NPT ensemble) during 75 ps. In the first 25 ps, velocities were rescaled every 0.1 ps, and in the second 25 ps, Langevin dynamics were used to maintain a constant temperature. Pressure control was introduced in the third 25 ps and in the production run using the Nosé–Hoover Langevin piston with a decay period of 500 fs. The harmonic restraints were gradually lifted (to 0.5, 0.25 and 0.05 kcal/(mol·Å^2^)) in the three equilibration stages.

### 4.2. Molecular Dynamics (MD) Simulations

#### 4.2.1. Unbiased MD Simulations

After equilibration, unbiased NPT production runs were performed for 60 ns. The integration time step was 2 fs, and coordinates were saved with a sampling interval of 2 ps. All covalent bond lengths involving hydrogen atoms were fixed using the ”SHAKE”algorithm [36].

Three independent MD simulations were carried out by assigning different initial distributions of starting velocities to the minimized systems.

#### 4.2.2. Adaptive Biasing Force (ABF) MD Simulations

**Figure 8:**
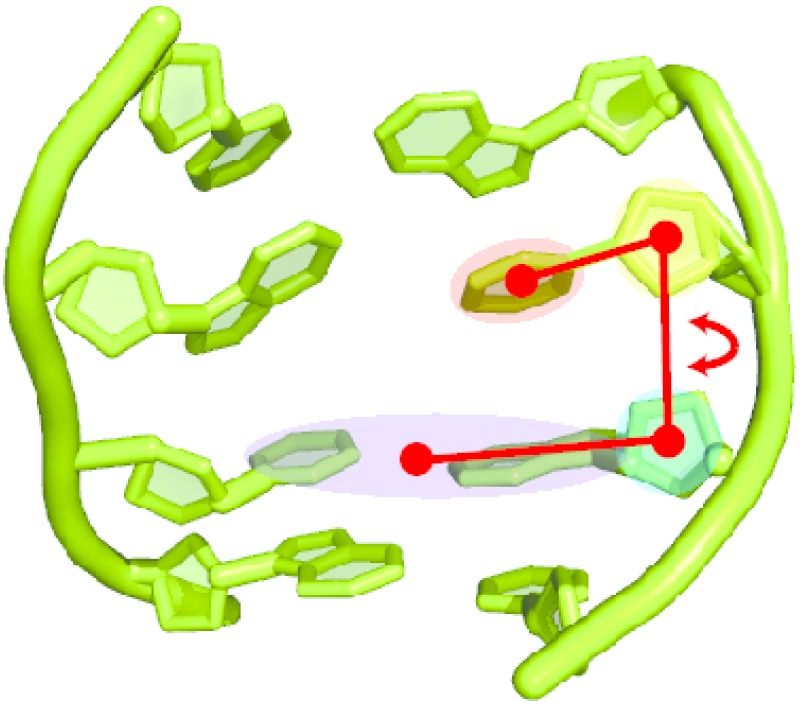
Definition of the reaction coordinate for the base flip simulations: the flip angle is a pseudo-dihedral between the centers of mass of the flipping base (red shade), the sugar moiety of the same nucleotide (yellow shade), the sugar moiety of the next nucleotide (green shade) and the base of the next nucleotide, plus the complementary base in the other DNA strand (blue shade).

For the simulation of the base flip, we applied the adaptive biasing force (ABF) method [37,38,39]. In ABF, the reaction coordinate is discretized into small bins. Sampling is carried out along the reaction coordinate in a continuous fashion. In each bin, samples of the instantaneous force acting along the reaction coordinate are accrued up to a certain threshold. If this threshold is reached, the adaptive biasing force is applied, so as to “drive” the system into the next bin. The reaction coordinate for the base flip has been defined as a pseudo-dihedral angle between the flipping base, the sugar moiety of the same nucleotide, the sugar of the next nucleotide and the base of the next nucleotide, plus the base and sugar of the opposing nucleotide downstream (see Figure 8). This definition of the flipping coordinate is the same as we had used in an earlier study [14] and is similar to the one proposed and applied in [21,40,41]. The potential of mean force (free energy profile) was obtained by discretizing the reaction coordinate between 10° and 180° into windows of a 2° width, and in each window, 2000 samples were collected before the bias was applied. For all systems, we carried out three ABF simulations of 60 ns in length, starting with different initial velocities.

#### 4.2.3. Analysis

For all analyzes (unbiased and ABF simulations), properties were evaluated for each run individually. Then, the averages and standard errors over the respective individual runs were calculated.

In the analyses of the unbiased MD simulations, the first 10 ns of each trajectory were not included.

The conformations of the G:oxC pairs in the DNA were characterized by calculating twelve helical parameters, six inter-base pair parameters (the three rotational parameters: roll, tilt and twist; and the three translational parameters: slide, rise and shift) and six intra-base pair parameters (rotation: buckle, propeller, opening; and translation: stagger, shear, stretch) that define the local DNA geometry. These parameters were measured using the Curve+ [42] suite of programs.

Hydrogen-bond occupancies were calculated as the ratio of the time when the hydrogen bond is formed to the total time of the trajectory. Two atoms are considered here to form a hydrogen bond if the acceptor-donor distance is <3.0 Å and the acceptor-hydrogen-donor angle is >135 °

Water accessibility of the (oxidized) methyl group was analyzed by calculating the solvent accessible surface area of that group. Solvent accessible surface areas have been computed by placing a probe sphere of radius *r*vdW+1.4 Å in contact with the atomic van der Waals sphere, both centered at the atom. The parts of the surface spheres where the center of the spherical probe can be placed without penetrating other atoms add up to the solvent accessible surface area [43].

### 4.3. Force Field Parameter Development

Bonded parameters were obtained from the ParamChem program [44,45,46,47,48].

For deriving the atomic charges of the oxidized methyl cytosine, we followed the procedure recommended in [45]. We have first geometry-optimized the oxidized bases (without sugar or phosphate groups) in vacuum at the Hartree–Fock/6-31G(d)level to a convergence criterion of 10^−^^6 ^a.u. using Gaussian G09 [49]. Then, a water molecule was added to the optimized structures at several different positions that allow for hydrogen bonds to be formed, and the relative orientation of the two molecules was optimized. The geometries and interaction energies were then used as a reference for fitting the charges of the oxidized bases.

Charges were fit applying a Monte Carlo procedure to minimize the error of water-base distances and interaction energies. As starting values of the atomic charges, we used the Mulliken charges obtained from the Hartree–Fock calculations. Only charges of the oxidized methyl group and the C5 host atom were adapted, so as to keep the new residues compliant with the existing CHARMM force field [45]. The Monte Carlo runs were repeated several times for 1000 steps each. The final charges are listed in the Supplementary (Tables S1 and S2).

### 4.4. Programs

All molecular images were generated with VMD (visual molecular dynamics) [50]. Structural analysis was performed using standard programs; Curve+ [51], Gromacs [52] tools and home-made scripts. The molecular dynamics simulations have been carried out using the program NAMD2.9 and applying the CHARMM27 force field. Simulations have been performed on the local Linux cluster of the physics department, on the ZEDATuniversity cluster (soroban), and using resources of the North-German Supercomputing Alliance (HLRN).

## 5. Conclusions

The different oxidized forms of methyl-cytosine investigated in this study show no intrinsic difference regarding their preference for a certain base-pair or base-step conformation. Moreover, the energy required to flip the methyl-cytosine base out of the DNA helix is similar in all four cases, indicating that the target base cannot be easily discriminated by the probability for base flip. Differences in the solvent accessibility and, in particular, different hydrogen bond patterns of the amino group N4 observed for the different forms of methyl-cytosine suggest a recognition mechanism in which the glycosylase enzymes attempt to form direct contacts. To what extent the imino forms of the cytosine bases that can form mismatch-like conformations and wobble-pair hydrogen bonding patterns contribute to recognition remains to be investigated.

## Supplementary Files

Supplementary File 1
