# Supplementary material for: Base Flip in DNA Studied by Molecular Dynamics Simulations of Differently-Oxidized Forms of Methyl-Cytosine
Source: Int J Mol Sci. 2014 Jul 3;15(7):11799–816. doi: 10.3390/ijms150711799 (PMC4139815; doi:10.3390/ijms150711799)
Supplement: Supplementary File 1 [file ijms-15-11799-s001.pdf]

Supplementary Information

Table S1. Interaction energies of cytosine, methyl cytosine and the oxidized methyl cytosine bases with guanine in Watson–Crick base pairs.

**Interaction Energy (kcal/mol)**	**Cyt**	**5mC**	**5hmC**	**5fC**	**caC**
	opt	opt	opt	opt	opt
CHARMM	−26.70	−26.84	−25.98	−23.77	−24.93
Hartree–Fock amino form	−25.52	−25.87	−24.43	−23.02	−28.46

Table S2. Charges and atom types for the oxidized forms of methyl-cytosine together with the available charges of cytosine and methyl-cytosine in the CHARMM force field. Newly developed charges are set in italics.

**CYT** [a]			**5MC** [a]			**5hmC**			**5fC**			**5caC**		
H5	HN3	0.07	C5M	CN9	−0.22	C5M	CT2	−0.12	C5M	CC	0.36	C5M	CC	0.62
						OX	OH1	−0.37	OX	OC	−0.56	OX1	OC	−0.76
			H5M1	HN9	0.09	HX	H	0.03	HX	HA	0.22	OX2	OC	−0.76
			H5M2	HN9	0.09	H51	HA	0.14						
			H5M3	HN9	0.09	H52	HA	0.25						
H6	HN3	0.17	H6	HN3	0.17	H6	HN3	0.17	H6	HN3	0.17	H6	HN3	0.17
N1	NN2	−0.50	N1	NN2	−0.50	N1	HN2	−0.50	N1	NN2	−0.50	N1	NN2	−0.50
H1	HN2	0.37	H1	HN2	0.37	H1	HN2	0.37	H1	HN2	0.37	H1	HN2	0.37
C6	CN3	0.17	C6	CN3	0.17	C6	CN3	0.17	C6	CN3	0.17	C6	CN3	0.17
C5	CN3	−0.13	C5	CN3D	−0.11	C6	CN3	−0.11	C5	CN3	−0.20	C5	CN3	−0.16
C2	CN1	0.52	C2	CN1	0.52	C2	CN1	0.52	C2	CN1	0.52	C2	CN1	0.52
N3	NN3	−0.66	N3	NN3	−0.66	N3	NN3	−0.66	N3	NN3	−0.66	N3	NN3	−0.66
C4	CN2	0.65	C4	CN2	0.65	C4	CN2	0.65	C4	CN2	0.65	C4	CN2	0.65
N4	NN1	−0.75	N4	NN1	−0.75	N4	NN1	−0.75	N4	NN1	−0.75	N4	NN1	−0.75
H41	HN1	0.37	H41	HN1	0.37	H41	HN1	0.37	H41	HN1	0.37	H41	HN1	0.37
H42	HN1	0.33	H42	HN1	0.33	H41	HN1	0.33	H42	HN1	0.33	H42	HN1	0.33

[a] Standard residues of the CHARMM force field.
